# Supplementary material for: Direct segmentation of cortical cytoarchitectonic domains using ultra-high-resolution whole-brain diffusion MRI
Source: Imaging Neurosci (Camb). 2024 Dec 20;2:imag-2-00393. doi: 10.1162/imag_a_00393 (PMC12315736; doi:10.1162/imag_a_00393)
Supplement: Supplementary Material A [file imag_a_00393-suppla.pdf]

Supplementary Material A

We evaluated the correspondence between the warping-based D99 cortical parcellation and direct MAP cytoarchitectonic segmentations and assessed their accuracy in identifying boundaries between cortical areas by comparing them with the corresponding histological images. Boundaries between cortical areas were manually delineated by an experienced neuroanatomist based on discontinuities in the laminar patterns observed in one or more histologically stained 2D coronal sections. During this process, the anatomist used the D99 atlas labels in standard space as an anatomical reference but was blinded to any subject-specific MRI-derived data including the MAP-MRI-derived parameter volumes and segmentations, as well as to the warped D99 atlas labels. The areal boundary demarcations were then superimposed on the matched coronal slice from the MTR volume to serve as a gold standard in the subsequent comparison with the MAP-based and warping-based segmentations. Since all dMRI data was co-registered to the structural MTR scan, the histologically defined areal boundaries can be directly compared to those derived using the MAP- and warping-based segmentations. In a few areas where the cortical geometry varied drastically through the coronal plane the boundaries were carefully adjusted to account for slight misalignments in the cortical ribbon geometry across corresponding coronal sections from different histological stains and/or MRI slices.

In a similar fashion we estimated areal borders using only the MAP-based segmentation. Specifically, we manually delineated boundaries radially across the cortical ribbon in regions with sharp, conspicuous laminar pattern discontinuities in 2D coronal slices from the MAP-based cytoarchitectonic segmentation volume (Fig. SA1, red lines). Finally, we superimposed both the histologically defined areal boundaries (Fig. SA1, yellow lines) and the MAP-defined borders ((Fig. SA1, red lines) onto the corresponding 2D coronal slice of the warped D99 cortical parcellation, thereby allowing for a direct and quantitative comparison of areal boundary estimates from all three approaches simultaneously (Fig. SA1).

As expected, for most cortical areas, the histological boundaries are more closely aligned with the corresponding areal borders segmented from the MAP parameters than with those of the warped D99 atlas segmentation. This is not surprising since discontinuities in laminar patterns of the MAP segmentation reflect intrinsic cytoarchitectonic contrast observable with high-resolution dMRI, whereas the warped D99 segmentation lacks cortical contrast relying primarily on extrinsic anatomical landmarks, such as the geometry of the cortical ribbon. Figs. SA1 A and B show the same regions from Figs. 7 and 8 in the manuscript, respectively, and include scale bars to allow readers to quantitatively estimate and compare distances between the areal borders estimated using the three methods. This direct comparison provides a more quantitative assessment of the segmentation accuracies but is still limited to 2D sections. Within a 2D section the orientations of the estimated areal borders from the three methods can differ. Consequently, the relative distances between the corresponding borders should be measured at a consistent cortical depth, e.g., the mid-cortical layer.

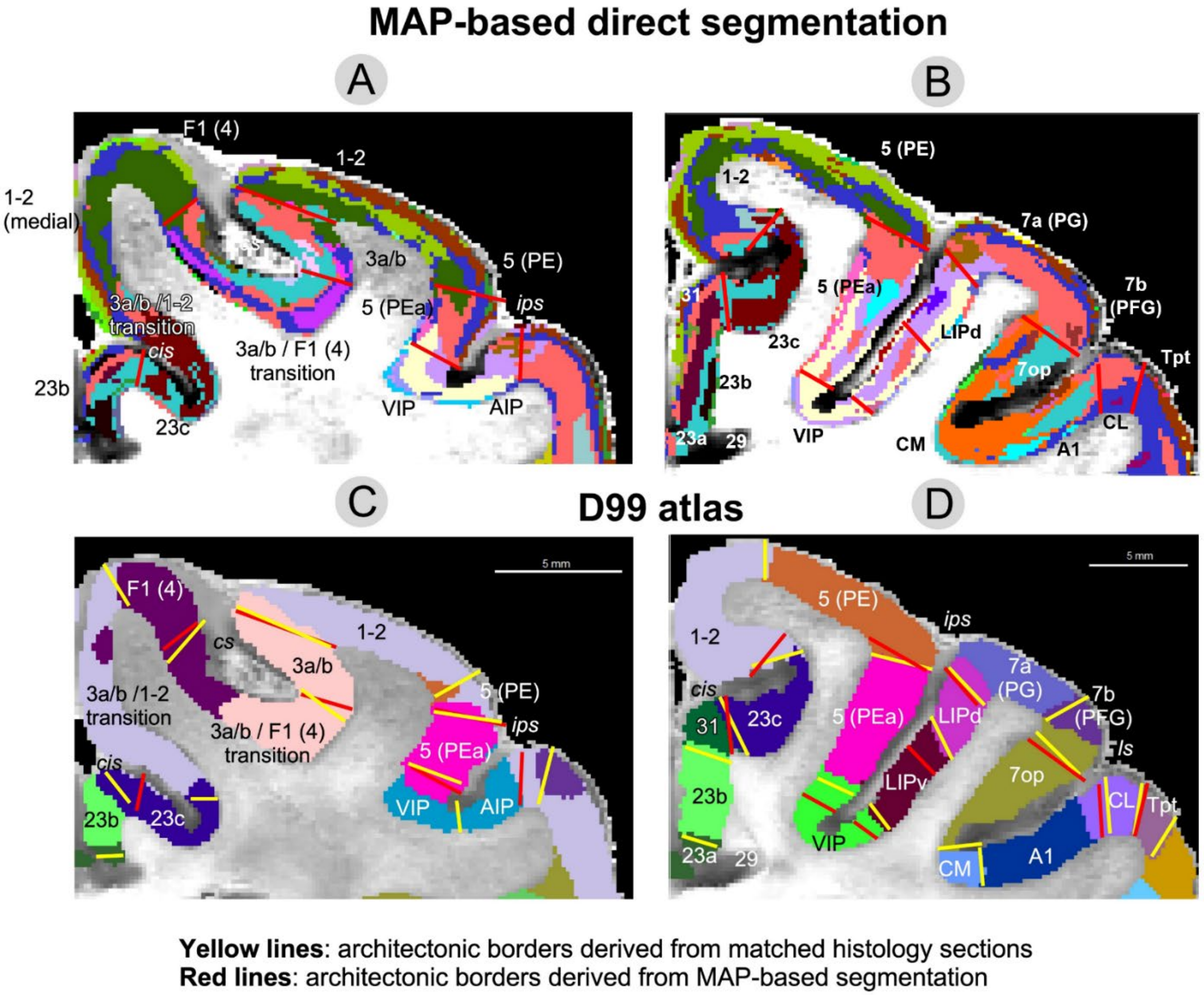

Figure SA1: Comparison of areal borders derived using histology (yellow lines), MAP-based segmentation (red lines), and the warped D99 atlas. In both figures C and D, the yellow lines represent areal borders manually drawn on matched histological coronal sections shown in Figs. 7 and 8 in the manuscript, respectively. The red lines represent areal borders manually delineated at transition regions between laminar patterns observed on the MAP-based segmentation (A and B). The areal borders correspond better with histology in the MAP-derived segmentation compared to the warped D99 segmentation. Differences between borders can be measured using the scale bar in each figure.
