# Supplementary material for: Direct segmentation of cortical cytoarchitectonic domains using ultra-high-resolution whole-brain diffusion MRI
Source: Imaging Neurosci (Camb). 2024 Dec 20;2:imag-2-00393. doi: 10.1162/imag_a_00393 (PMC12315736; doi:10.1162/imag_a_00393)
Supplement: Supplementary Material B [file imag_a_00393-supplb.pdf]

Supplementary Material B

To gain deeper insight into the relative utility of DTI- vs MAP-derived microstructural parameters for direct cortical cytoarchitectonic segmentation we extended our analysis to re-produce Figs. 5-8 using only DTI parameters. Specifically, we repeated the GMM analysis using 14 clusters and an input dataset containing the DTI parameters FA, RD, and AD, followed by the same 3D morphological filtering and label matching steps used in our original analysis with MAP input parameters PA, NG, RTAP, RTPP (results shown in manuscript). The BIC values for the left and right hemispheres were 8.4*10^6 and 8.5*10^6, respectively, for the DTI input parameters and 9.8*10^6 and 1.0*10^7, respectively, for MAP input parameters. Note that while GMM analysis using the DTI parameters yielded a lower BIC than using the MAP parameters, the resulting segmentation showed a more fragmented appearance of cortical layers (Fig. SB1A-B, red arrows; vs. Fig. 5A-B in the manuscript). Moreover, in the DTI-based segmentation, the assignment of clusters shows an asymmetry between the left and right hemispheres (Fig. SB2 vs. Fig. 6 in the manuscript) suggesting inconsistencies between the sizes, shapes, and locations of cytoarchitectonic clusters obtained in the left and right hemispheres.

A possible explanation is that the DTI model may not accurately capture diffusion anisotropy in cortical layers with different mixtures of radial and tangential components (Avram, Saleem et al. 2022), yielding estimates of FA that may potentially reflect biases due to the curvature of the cortical ribbon. Consequently, some regions segmented based on DTI parameters may not accurately reflect intrinsic tissue microstructural and architectural features. For example, in Fig. R7C, the border between areas 1-2 and 3a/b, the DTI-based segmentation does not identify the laminar pattern discontinuity observed both in the MAP-based segmentation (Fig. 7C in the manuscript) or the warping-based parcellation (Fig. SB3D), suggesting that extrinsic factors such as cortical geometry can bias the DTI-based cytoarchitectonic parcellation. In Fig. SB3, the borders between areas 1-2 and 3a/b, areas F1(4) and 3a/b, or between VIP and AIP align more accurately with the laminar pattern discontinuities from the MAP-derived segmentation (Fig. 7C) than with those from the DTI-derived segmentation (Fig. SB3C).

On the other hand, MAP parameters quantify features of the diffusion propagator comprehensively (not just its Gaussian approximation) reducing bias on extrinsic cortical properties (e.g., sulcal geometry) and providing a more faithful delineation of cytoarchitectonic regions that correlates well with histology (Avram, Saleem et al. 2022). The discrepancies between the DTI- and MAP-based microstructural parameters (e.g., FA vs. PA) are relatively limited in our study due to the high spatial resolution of our data (200μm). At smaller voxel sizes, the decreased tissue architectural heterogeneity within the voxel reduces the discrepancies between the tensor model and the MAP signal representation. In many areas, the DTI-based (Fig. SB4C) and MAP-based segmentation (Fig. 8C) reveal similar discontinuities in the segmented laminar patterns which correspond well with those observed in the PV (E) and SMI-32 (F) matched histological slices.

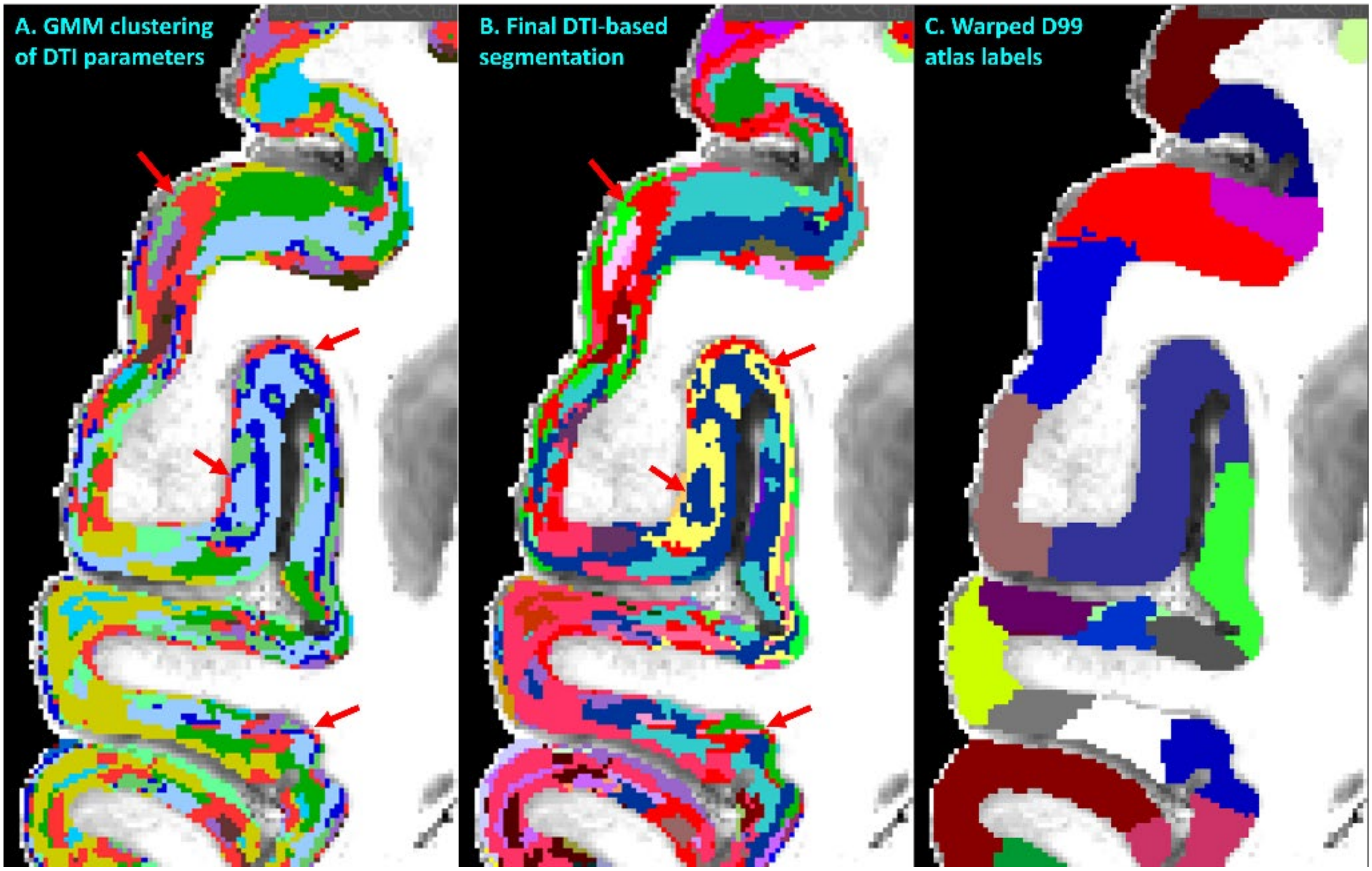

Figure R5: DTI parameter values (FA, RD, and AD) from all cortical voxels in each hemisphere were segregated using GMM in 14 distinct clusters (A). The resulting image was processed with a 3D morphological filter to merge small isolated spatial components/islands and uniquely relabel all spatially disjoint components. The final DTI-based segmentation (B) was obtained by matching labels between the left and right hemispheres. Unlike the MAP-based segmentations (Figs. 5A and 5B in the manuscript), the DTI-based segmentations (A and B) show a more fragmented appearance of cortical layers (red arrows) likely due to potential biases in the measurement of diffusion anisotropy introduced by the tensor model. The same region from the axial slice from Fig. 5 in the manuscript is shown.

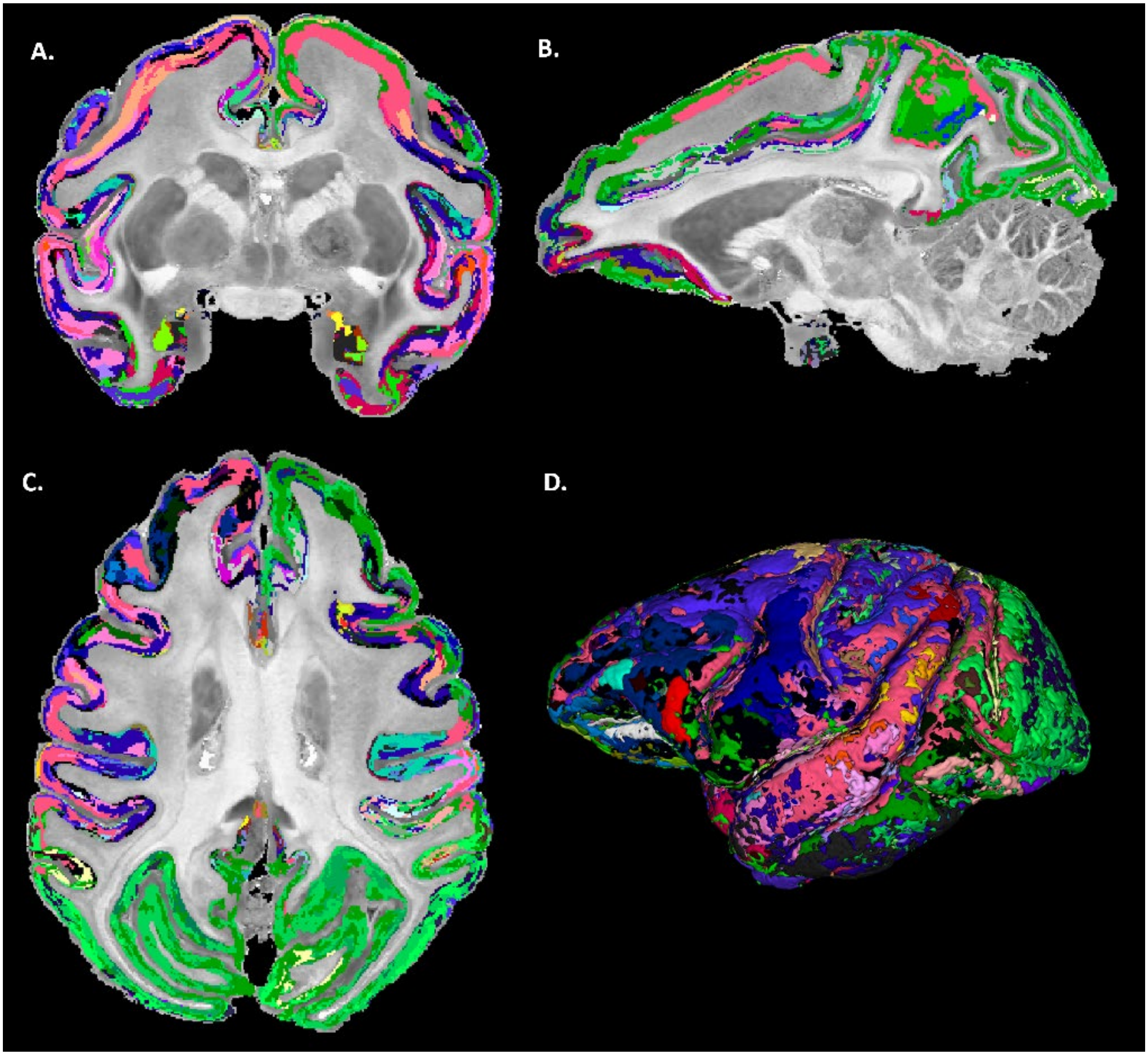

Figure R6: Final DTI-based cytoarchitectonic cortical domain segmentation after matching the cytoarchitectonic labels across the left and right hemispheres. Unlike the MAP-based segmentation (Fig. 6 in the manuscript), the left and right hemispheres show a certain level of asymmetry suggesting larger differences between the sizes, shapes, and locations of clusters computed separately in the two hemispheres. These interhemispheric differences likely arise due to increased fragmentation within individual layers in the DTI-based segmentation.

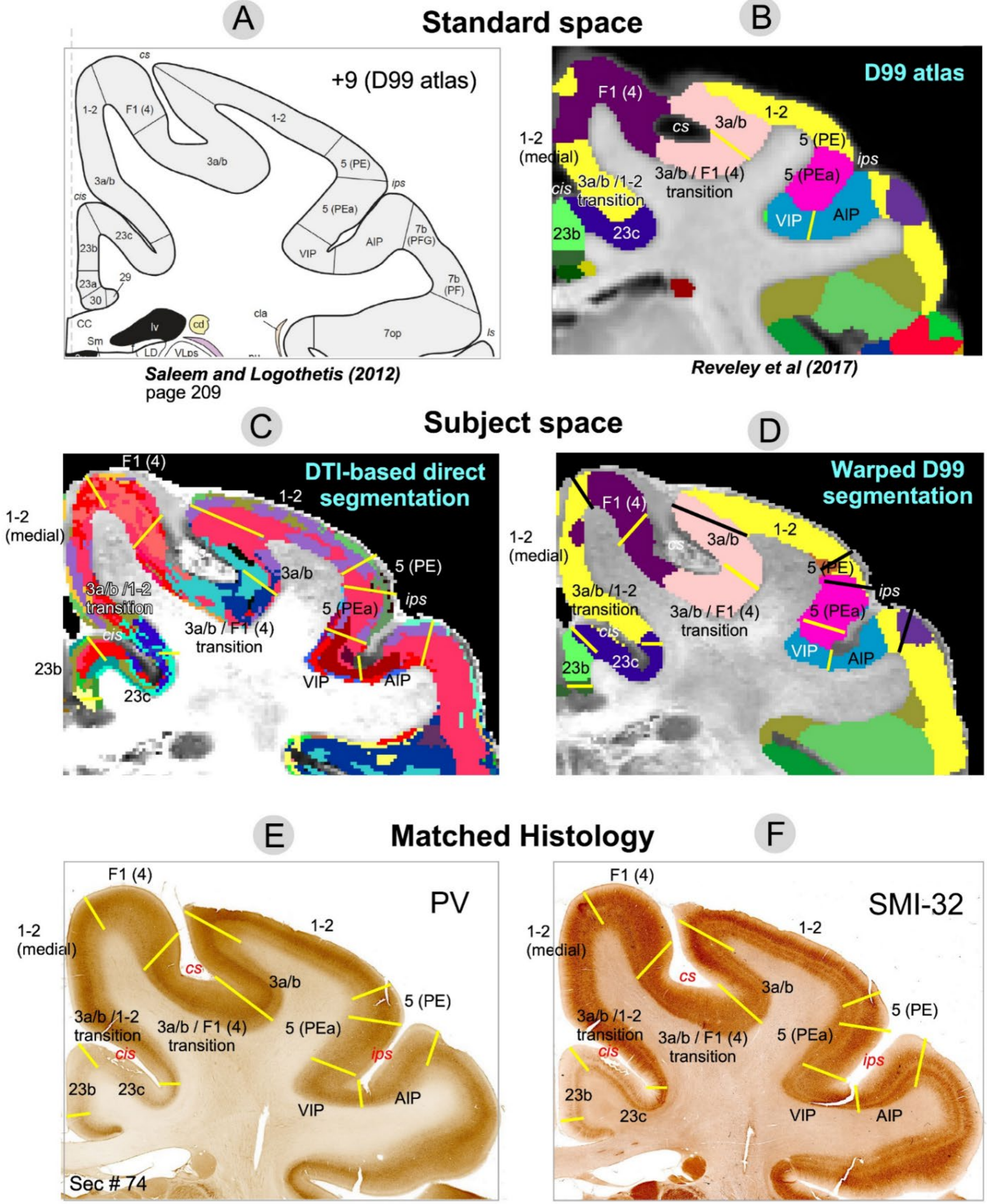

Figure R7: Comparison of warped D99 and the direct DTI-based segmentations with two histological stains in the same brain region shown in Fig. 7 in the manuscript. Some the transition regions between laminar patterns in the DTI-derived segmentation correspond well both with the histology and the MAP-derived segmentation. The borders between areas 1-2 and 3a/b, areas F1(4) and 3a/b, or between VIP and AIP align more accurately with the laminar pattern discontinuities from the MAP-derived segmentation (Fig. 7C) than with those from the DTI-derived segmentation (C).

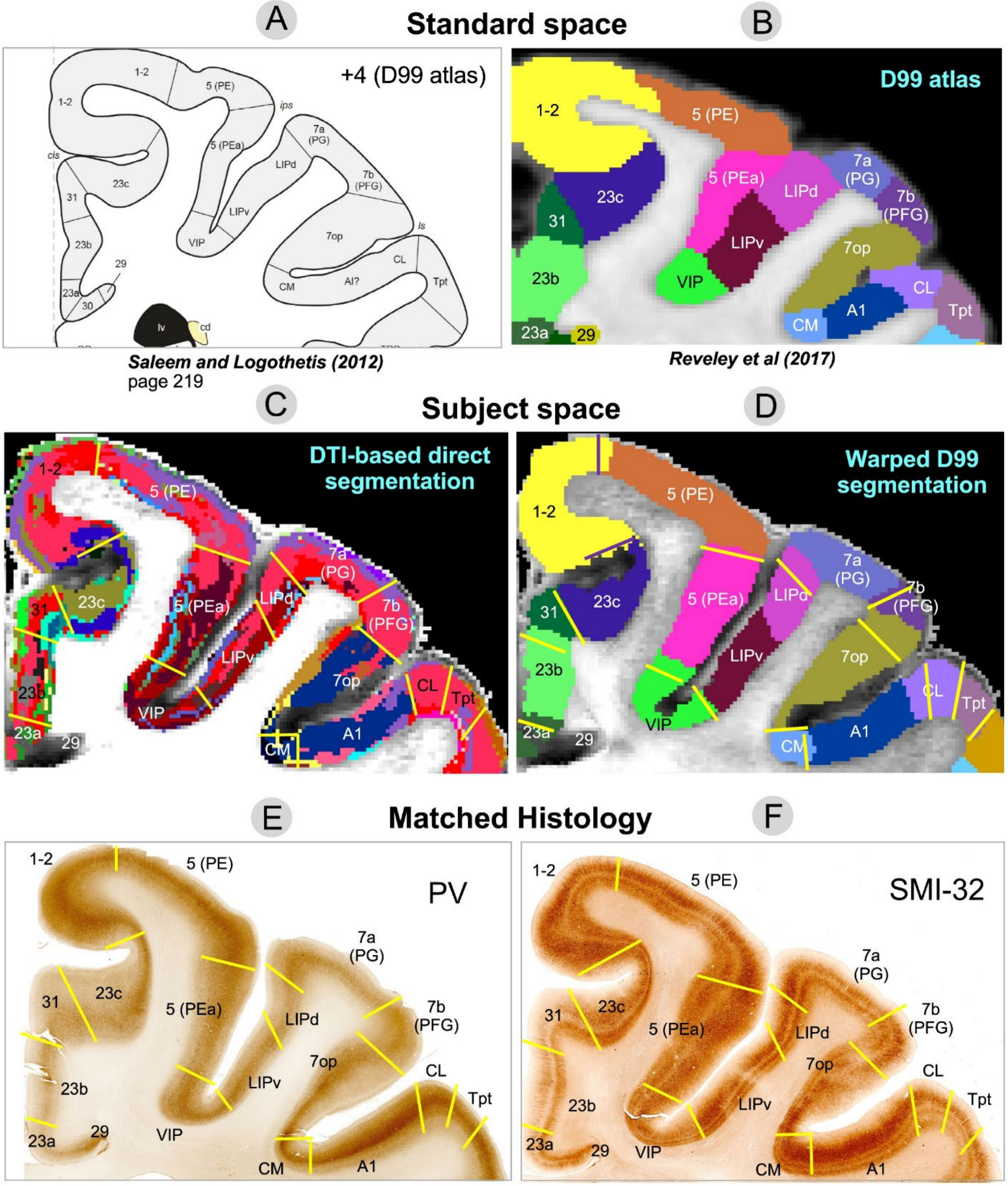

Figure R8: Comparison of warped D99 and direct DTI-based segmentations with two histological stains in the same brain region shown in Fig. 8 in the manuscript. The transition region between areas 5(PE) and 5(PEa) is not as sharp in the DTI-derived (C) in the MAP-derived segmentation (Fig. 8C). However, in many areas, the DTI-based (C) and MAP-based segmentation (Fig. 8C) reveal similar discontinuities in the segmented laminar patterns which correspond well with those observed in the PV (E) and SMI-32 (F) matched histological slices.

References

Avram, A. V., K. S. Saleem, M. E. Komlosh, C. C. Yen, F. Q. Ye and P. J. Basser (2022). "High-resolution cortical MAP-MRI reveals areal borders and laminar substructures observed with histological staining." NeuroImage **264**: 119653.
